# Exposure to sublethal concentrations of methoxyfenozide disrupts honey bee colony activity and thermoregulation

**DOI:** 10.1371/journal.pone.0204635

**Published:** 2019-03-28

**Authors:** William G. Meikle, Vanessa Corby-Harris, Mark J. Carroll, Milagra Weiss, Lucy A. Snyder, Charlotte A. D. Meador, Eli Beren, Nicholas Brown

**Affiliations:** Carl Hayden Bee Research Center, USDA-ARS, Tucson, AZ, United States of America; Institut Sophia Agrobiotech, FRANCE

## Abstract

Methoxyfenozide is an insect growth regulator (IGR) commonly used in agriculture to simultaneously control pests and preserve beneficial insect populations; however, its impact on honey bees in not fully understood. We conducted field and laboratory experiments to investigate bee health in response to field-relevant concentrations of this pesticide. Significant effects were observed in honey bee colony flight activity and thermoregulation after being exposed over 9 weeks to supplemental protein patty containing methoxyfenozide. Compared to bee colonies in the control group, colonies fed pollen patty with 200 ppb methoxyfenozide (as measured by residue analysis) had: 1) a significantly reduced rate of weight loss due to forager departure in the morning; and 2) higher temperature variability during the winter. Colonies in the 100 ppb (as measured by residue analysis) treatment group had values between the 200 ppb group and control for both response variables. The dusk break point, which is the time associated with the end of forager return, differed among all treatment groups but may have been confounded with direction the hives were facing. Bee colony metrics of adult bee mass and brood surface area, and measurements of bee head weight, newly-emerged bee weight, and hypopharyngeal gland size were not significantly affected by methoxyfenozide exposure, suggesting that there may be significant effects on honey bee colony behavior and health in the field that are difficult to detect using standard methods for assessing bee colonies and individuals. The second experiment was continued into the following spring, using the same treatment groups as in the fall. Fewer differences were observed among groups in the spring than the fall, possibly because of abundant spring forage and consequent reduced treatment patty consumption. Residue analyses showed that: 1) observed methoxyfenozide concentrations in treatment patty were about 18–60% lower than the calculated concentrations; 2) no residues were observed in wax in any treatment; and 3) methoxyfenozide was detected in bee bread only in the 200 ppb treatment group, at about 1–2.5% of the observed patty concentration.

## Introduction

Honey bee colonies are frequently exposed to agrochemicals, including many different classes of insecticides, among them insect growth regulators (IGRs) [1, 2]. Broadly speaking, IGRs interfere with insect growth and development in their target pest species [3]. The IGR methoxyfenozide is an ecdysone receptor agonist that binds the ecdysteroid receptor and activates the ecdysteroid signaling pathway [4, 5]. Unlike the ecdysteroid hormone that precisely controls larval development by binding to the receptor temporarily, ecdysteroid agonists bind irreversibly, disrupting the expression of genes involved in cuticle development, sclerotization, and ecdysis [5, 6]. Methoxyfenozide specifically targets lepidopterans and research shows that it has high affinity for the lepidopteran ecdysteroid receptor that is not seen in other insect orders (reviewed in [4, 5]); the binding specificity of methoxyfenozide to the *Apis mellifera* ecdysteroid receptor has not been determined. Fewer non-target effects makes methoxyfenozide an attractive, targeted solution for controlling pests while preserving beneficial insect populations. Methoxyfenozide is registered in more than 50 countries for use in a variety of crops, including those pollinated by honey bees. Methoxyfenozide use has increased 15-fold between 2001 and 2015, from ~30,000 to ~450,000 pounds annually [7], mostly in orchards, to control lepidopteran pests like the navel orangeworm, *Amyelois transitella* (Walker) [8].

Reported toxicity of methoxyfenozide to young worker bees is low, with an acute toxicity LD_50_ greater than 100 ug/bee [9] and, when formulated with spinetoram, an oral toxicity LC_50_ of about 712 mg/L (ppm) for newly emerged workers [10]. Nevertheless growers have been advised to avoid spraying during bloom because the impact of methoxyfenozide on earlier life stages, older workers, or on colonies is not fully understood [11]. Despite these spray recommendations, methoxyfenozide has been detected in commercial colonies in bees (9–21 ppb) and hive materials including wax (80–495 ppb), pollen (35–128 ppb), and honey (3 ppb) [2, 12, 13]. In a recent study, methoxyfenozide exposure decreased forager survival at field-relevant concentrations [14].

Other IGRs cause delayed sublethal effects in adult honey bees after larval exposure. Larval bees exposed to the juvenile hormone analog pyriproxyfen in pollen patty at 321 ppb showed increased deformities as adults, reduced adult survivorship and exposed adults had difficulties integrating into the general adult population; fewer effects were observed at the lower concentration of 129 ppb [15]. The impact of methoxyfenozide on honey bee colonies is not fully understood. Although methoxyfenozide is marketed as a bee safe pesticide due to its specificity to certain pests, it is nonetheless possible that honey bees are negatively impacted simply due to the energetic cost of detoxifying an exogenous chemical [16–18]. Pesticide exposure can lead to increased expression of stress response and detoxification genes [19], but expression patterns vary [20]. It is unclear whether detoxification, *per se*, is stressful to honey bees, although metabolomic analyses of bees exposed to plant secondary toxins suggest that detoxification of other compounds is costly [16]; nurse-aged bees exposed to some classes of pesticides have reduced hypopharyngeal glands [20, 21], a pattern observed with other stressors [22].

Effects that are difficult to detect on the individual level may be detected on the colony level [23]. Continuously monitoring weight and internal hive temperature of honey bee hives has provided information on bee colony growth and activity [24–26]. In those studies, continuous weight and hive temperature data were detrended by subtracting the 25-hour running average from the raw data. Running average weight data provided information on longer-term colony growth while the detrended within-day data were modeled using sine curves to yield information on foraging activity and success. Similarly, the average and detrended hive temperature data were related to capped brood levels [24]. These approaches were used to detect colony-level treatment effects of sublethal exposure of the neonicotinoid pesticide imidacloprid on flight activity and internal hive temperature control (colony thermoregulation) [27]. In another approach, continuous hive weight data were detrended by subtracting the value at midnight, rather than the running average, from each of the values of that day until the next midnight [28]. In that approach, a piecewise regression model was fitted to single-day datasets in the original time scale using an R function [29] based on a bootstrapping method [30]. Several parameters of the piecewise regression provide biological or behavioral interpretations, such as break points around dawn and dusk reflecting the beginning and ending of the daily active periods for the hive [28].

To more fully address the possible consequences of methoxyfenozide exposure on honey bees, we conducted replicated field and laboratory investigations of bee health in response to field-relevant concentrations of this pesticide. In two different years, we mixed methoxyfenozide into pollen patty and fed it to colonies. Colony parameters such as brood area, adult weight, foraging activity, and hive temperature were measured. We find that methoxyfenozide does not exert a massively negative effect on honey bees, but that small differences among hives in foraging activity and hive temperature regulation appear to be exacerbated in the high-concentration treatments compared to the control.

## Materials and methods

### 1. Preparation of pollen supplement

A 50 mg/ml stock solution of methoxyfenozide (Millipore-Sigma CAS no. 161050-58-4, product no. 32507) was prepared in acetone. 18 kg of pollen patty supplement was prepared in 1.5 kg batches in a stand mixer at a ratio of 1:1:1 corbicular pollen (Great Lakes Bee Co.):granulated sugar:drivert sugar (Domino Foods). For the 500 ppb treatment patty, hereafter the “high” treatment, 15 μL of the stock solution of methoxyfenozide was added to 235 μL of acetone yielding a 3 mg/ml solution. 250 μL of this 3 mg/ml solution was added to 130 ml of water and then thoroughly mixed with 1.5 kg of patty. Similarly, the 125 ppb treatment patty, hereafter the “low” treatment, was prepared by adding 3.75 ul of the stock solution to 246.25 ul acetone yielding a 0.75 mg/ml solution. 250 μL of this 0.75 mg/ml solution was added to 130 mL water and thoroughly mixed into 1.5 kg of patty. All of the diet was prepared at the same time for the Fall 2016 experiment. The diet was divided into 100 g patties and stored at -20°C until the supplement was fed.

Pollen supplement for Fall 2017 and Spring 2018 experiments was prepared in a similar manner except that the methoxyfenozide stock solution was prepared to 6 mg/ml in acetone. 125 μL of that solution was then mixed with 100 mL water for a concentration of 0.75 mg/100 mL and applied to 1.5 kg supplement for a concentration of 500 ppb. Likewise, 31 μL of stock solution was diluted with 93 μL acetone and mixed with 100 mL water per 1.5 kg diet for a concentration of 125 ppb. Control patties were treated with 125 μL pure acetone and 100 mL water. Patties were divided and stored in the same manner. Patty samples (3 g) were submitted to the Laboratory Approval and Testing Division, Agriculture Marketing Service, USDA (LATD), Gastonia, NC, to determine methoxyfenozide concentrations.

### 2. Fall 2016 field experiment

In August 2016, eighteen honey bee colonies were selected from apiaries at or near the Carl Hayden Bee Research Laboratory, USDA-ARS, Tucson, AZ (32°16'30.17"N, 110°56'28.52"W) and the Santa Rita Experimental Range (SRER) (31°46'38.08"N, 110°51'47.39"W) and moved to a single site at SRER in August, 2016. Colonies had Cordovan-Italian queens (C.F. Koehnen & Sons, Glenn, CA) and were housed in two painted, 10-frame wooden Langstroth deep boxes fitted with migratory wooden lids (Mann Lake Ltd, Hackensack, MN). Several frames of drawn comb and foundation comb were placed in each hive. Colonies were 6–18 months old at the start of the experiment and all had queens from 2016. The SRER apiary was provided with a permanent water source. The apiary was surrounded by native, unmanaged plants, particularly mesquite (*Prosopis* spp.), creosote (*Larrea* spp.), cactus (mainly *Opuntia* spp.) and wildflowers. No commercial agriculture exists within a 10 km of the apiary. Hives were placed on stainless steel electronic scales (TEKFA model B-2418 and Avery Weigh-Tronix model BSAO1824-200) (max. capacity 100 kg) connected to a solar power source and to 16-bit dataloggers (Hobo UX120-006M, Onset Computer Corporation) that were set to record weight every 5 minutes. Hives were arranged in groups of 4–5 and each group was placed around the steel box housing the dataloggers and power source. To reduce drift among hives, hive entrances faced outward from the box in different directions (N, S, E, W with two hives facing SE). Each group was 2 or more meters from neighboring groups. Prior to placing the hives on the scales, and at the end of the experiment, all scales were calibrated using commercial scale weights. Changes in calibration parameters over time were negligible. The system had an overall precision of approximately ±20 g. On the same day, a hive temperature sensor (iButton Thermochron, precision ±0.06°C) enclosed in plastic tissue embedding cassettes (Thermo Fisher Scientific, Waltham, MA) was stapled to the center of the top bar on the 5th frame in the lower box of each hive and set to record every 30 min.

On 29–30 August, 2016, hives were given a full pre-treatment assessment (see [26, 31]). Briefly, the hive was opened after the application of smoke, and each frame was lifted out, gently shaken to dislodge adult bees, photographed using a 16.3 megapixel digital camera (Canon Rebel SL1, Canon USA, Inc., Melville, NY), weighed on a portable scale (model EC15, OHaus, 15 kg max. cap.), and replaced in the hive. Frames were removed and replaced sequentially. During this first assessment (but not subsequent assessments), all hive components (i.e. lid, inner cover, box, bottom board, frames, entrance reducer, internal feeder) were also shaken free of bees and weighed to yield an initial mass of all hive components. At the initial inspection, 3–5 g of wax were collected from each hive into 50 ml centrifuge tubes and stored at -80°C; samples collected in September, prior to treatment, were pooled and subjected to a full panel analysis for residues of 192 pesticides and fungicides, from all major classes, by LATD. Samples from later assessments were pooled within treatment group and subjected only to methoxyfenozide residue analysis.

After the initial hive assessment, the total adult bee mass (weight of the entire adult bee population per colony) was calculated by subtracting the combined weights of hive components obtained in the pre-treatment assessment from the total weight of bees and hive materials recorded the midnight prior to the inspection. The area of sealed brood per frame was estimated from the photographs using ImageJ version 1.47 software (W. Rasband, National Institutes of Health, USA). Three treatment groups were then constructed by dividing the colonies evenly into low, medium and high groups based on adult bee mass and then selecting 2 hives from each of the three adult bee mass groups, in order to harmonize the average and variance of adult bee masses among treatment groups (Table 1). One treatment regime, low (125 ppb), high (500 ppb) or control, was then randomly assigned to one of the three treatment groups. Each hive was given 100 g pollen patty once to twice a week with the appropriate concentration starting on 2 September, continuing weekly for 9 weeks until 28 October (1300 g treatment pollen patty total per hive). Consumption of the patty was measured by weighing any patty (wet weight) that remained after one week. All colonies were treated against Varroa mites by placing Apivar strips (Veto-Pharma) in the hives in early September and removing them 6 weeks later. Mite levels were monitored after the treatment by placing sticky boards under the hives for 3 days and calculating the average daily mite fall. Hives were assessed on 2 November, 2016 (first post-treatment assessment, 61 d after initial treatment). Each colony was then given 3 kg of 1:1 sugar syrup and frame of capped honey on 10 November because of low food stores. Colonies were assessed and sampled for the final time on 30 January, 2017, (2^nd^ post-treatment assessment) to determine the long-term effects of methoxyfenozide exposure on overwintering.

**Table 1:** Adult bee masses and brood surface areas for the Fall 2016, Fall 2017 and Spring 2018 field experiments. High, low and control refer to methoxyfenozide treatment groups.

	Adult bee mass (g)			Capped brood surface area (cm^2^)		
Dates	high	low	control	high	low	control
Fall 2016:						
Pre-treatment 30 Aug. 2016	2254 ±234	2166 ±141	2202 ±204	1972.4 ±436.0	2120.6 ±220.7	2475.5 ±291.7
Post treatment 2 Nov. 2016	1498 ±330	1550 ±153	1633 ±139	498.0 ±134.6	583.2 ±53.1	546.6 ±97.7
Post winter 1 Feb. 2017	777 ±296	782 ±224	1352 ±107	3.7 ±0.6	9.1 ±8.0	11.6 ±7.9
Fall 2017:						
Pre-treatment 13 Sep. 2017	1591 ±208	1641 ±251	1595 ±141	630.1 ±158.3	426.4 ±112.5	756.7 ±98.3
Post treatment 15 Nov. 2017	1496 ±224	1563 ±170	1434 ±83	210.6 ±33.1	274.9 ±38.4	269.4 ±53.0
Post winter 13 Feb. 2018	920 ±150	1010 ±230	810 ±90	320.1 ±125.4	412.8 ±96.2	496.7 ±153.6
Spring 2018:						
Pre-treatment 18 Apr. 2018	1453 ±416	1211 ±422	780 ±136	2613.8 ±640.1	1607.2 ±413.6	1055.6 ±245.7
Post treatment 23 May 2018	1553 ±491	1054 ±351	534 ±162	1292.7 ±259.2	1110.6 ±169.9	477.3 ±167.7

### 3. Fall 2017 and Spring 2018 field experiments

The experiment described above was repeated in 2017. In April, 2017 twenty colonies were started from packages from the same supplier as the previous year and installed in SRER at a site about 2 km away from the Fall 2016 site. Colonies were initially placed in single deep hive boxes, containing frames of drawn and foundation comb, and maintained with new Cordovan-Italian queens from the same queen supplier as the first year. Colonies were fed supplemental pollen patty in the spring, and 12 kg 1:1 sugar syrup between May and early September. As in the previous year, hives were placed around the equipment boxes, facing outward in a NE, NW, SE or SW direction. On 13 September 2017, full pre-treatment hive assessments were conducted on all colonies and 18 colonies selected for the study. Second deep boxes were added to 6 of the colonies because of their size; only temperature data from the bottom box were used. As was done the previous year, colonies were divided into low, medium, and high adult bee mass groups and three hive treatment groups constructed by selecting, for each treatment group, 2 hives from each adult bee mass group. As in the previous experiment, each colony was given 100 g treatment pollen patty twice per week beginning on 22 September and continuing for 8 weeks until 9 November (48 d after initial treatment, 1600 g treatment pollen patty total). Three supplemental feedings of 3 kg of 1:1 sugar syrup were provided to each colony during the treatment period. Colonies were treated against Varroa in the same manner and at about the same time period as the previous experiment. The 1^st^ post-treatment hive assessments were conducted on 15 November. Smaller colonies were reduced to single boxes for overwintering on 16 November 2017. The 2^nd^ post-treatment hive assessments were conducted on 13 February 2018. From 1 to 2 g of bee bread was collected from each hive at each assessment. As with the wax samples for the Fall 2016 experiment, samples collected in September, prior to treatment, were pooled and subjected to a full panel of residue analyses while samples from later assessments were pooled within treatment group and subjected only to methoxyfenozide residue analysis. Samples of protein patty from each treatment were also analyzed for methoxyfenozide concentration. In addition, newly emerged bees (NEBs) were also sampled by pressing an 8 cm x 8 cm x 2 cm mesh queen cage into a section of capped brood, then returning the following day to collect NEBs that had emerged within the cage over the previous 24 h. The NEBs were then placed in a 50 mL centrifuge tube, frozen on dry ice, and stored at -80°C. At the laboratory, up to 10 bees per hive (depending on how many were collected) per assessment date were placed in Eppendorf tubes, weighed, dried for 72 h at 60°C, then re-weighed to determine average wet and dry weight per bee.

The hives used in the Fall 2017 experiment were retained in the same treatment groups and, starting on 8 March, fed 100 g pollen patty twice weekly for 6 weeks until 13 April (42 d after initial treatment, 1200 g treatment pollen patty total). All hives were given 4 L supplemental sugar syrup on 30 March. Hives were evaluated on 18 April (1^st^ post-treatment assessment) and again on 24 May (2^nd^ post-treatment assessment). Bee bread and NEBs were sampled at each assessment. As above, temperature data from the bottom box were used in the analysis.

### 4. Adult bee head weights from Fall 2017 experiment

Head weight was measured on samples of nurse-aged bees collected from all colonies after the November 2017 post-treatment hive evaluation. Nurse-aged bees visiting cells containing larvae for a period of ≥5 seconds were collected. The captured bees were immediately flash frozen in the field and were maintained at -80°C until their heads were weighed. To obtain head weights, each head was thawed and weighed using a using a Sartorius CP2P microbalance at a resolution of 0.01 mg (see [32]).

### 5. Hypopharyngeal gland size of nurse-aged workers fed methoxyfenozide

In order to assess whether oral methoxyfenozide exposure affected nurse-aged bee health, HPG size was measured in caged nurse-aged bees fed either 1000 ppb of methoxyfenozide in 30% sugar syrup or a control treatment of methoxyfenozide-free sugar syrup. A comparatively high concentration was chosen to increase the probability of a measurable effect. NEBs emerged overnight from brood frames taken from three colonies in a temperature-controlled dark room (32–34°C, 30–40% relative humidity). The next morning, the NEBs were distributed among eight cages to a density of 100 bees per cage. Cage dimensions were 11.5 x 7.5 x 16.5 cm, with narrow sides, top and base made of Plexiglas and the broad sides and floor made of 3mm aperture galvanized steel mesh. A plastic 30 mL bottle for distilled water and a plastic 30mL bottle for syrup, each with small holes in the lid, were inverted and placed over holes on the top of each cage. Four cages were provided with 1000 ppb methoxyfenozide in 30% sucrose syrup and four cages with 30% sucrose syrup without the pesticide as controls. All cages were provided with pollen patty, 1:1:1 sucrose (100% table sugar; Domino Foods, Inc.): Drivert sugar (8% sucrose and fructose + 92% sucrose; Domino Foods, Inc.): natural pollen (Bulkfoods.com) *ad libitum*. The caged bees were maintained at 34°C and 30–40% relative humidity. At 8d after emergence, 10 bees per cage were flash frozen and maintained at -80°C until their HPGs were dissected and measured (see [33]). Between 10 and 12 HPG acini were measured for each gland to obtain an average HPG acinus size for each bee.

### 6. Data analysis

#### 1. Hive assessment, patty consumption, NEB weights, nurse bee head weights and HPG size

Adult bee masses, capped brood surface areas and average NEB dry weight per hive were compared among treatments across sampling occasions using repeated-measures MANOVA (SAS version 9.4) with treatment, experiment and day as main factors, all 2-way interactions, and with pre-treatment adult bee mass as a covariate to control for pre-existing colony differences. Per colony patty consumption, NEB weights and nurse bee head weights were analyzed using repeated-measures MANOVA with treatment was the main effect and hive was a random effect. Caged-bee data were analyzed using ANOVA, with treatment as a fixed effect and cage replicate as a random effect. Post hoc contrasts with the Bonferroni correction for multiple comparisons were reported for significant treatment effects.

#### 2. Hive weight

**Fig 1:**
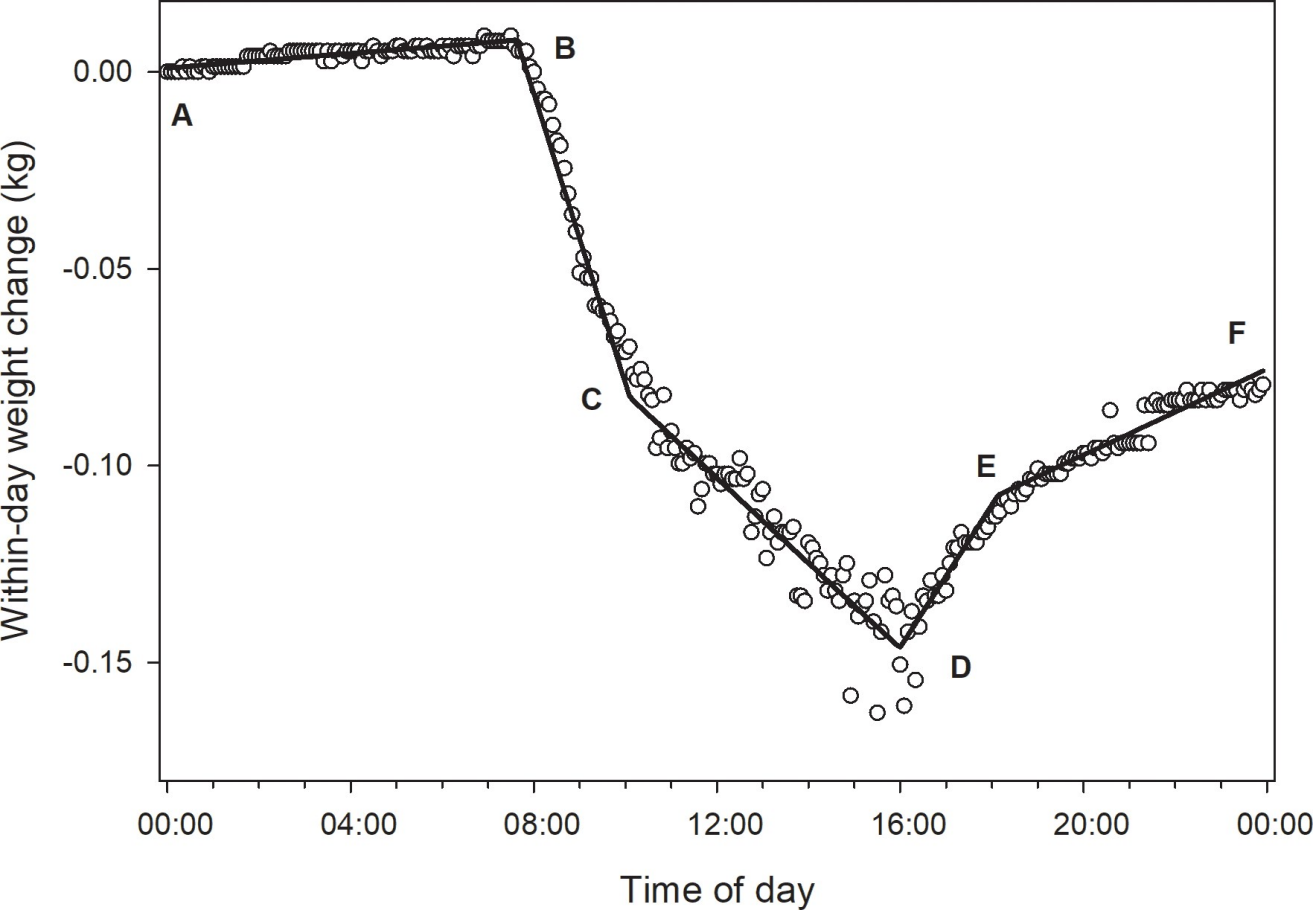
Within-day hive weight data and associated fitted piecewise regression. Data were obtained from a hive in the control group on 24 November 2017 during a forage dearth. **1.Point A:** First weight measure at midnight or shortly thereafter; **2.Segment AB:** Inactive period in the early morning; hive weight change is likely due to bee respiration and changes in the moisture content of nectar, pollen and wooden hive parts; **3.Point B:** Bee departure at beginning of active period, usually close to dawn - 1^st^ break point; **4.Segment BC:** Active period usually showing hive weight loss due to far greater numbers of departing bees compared to returning bees- “departing slope”; **5.Point C:** Point at which rate of hive weight loss changes, likely due to higher ratio of returning bee mass compared to departing bee mass; **6.Segment CD:** Continued increase in hive weight due to forager return; **7.Point D:** Point at which mass of returning foragers, including nectar and pollen as well as bee weight loss due to respiration, equals the mass of departing bees plus weight loss due to drying and respiration in the colony; **8.Segment DE:** Increasing hive weight due to returning foragers; **9.Point E:** Return of bees to the hive around dusk is completed–last break point; **10.Segment EF:** Inactive period with hive weight change driven mainly by respiration and changes in ambient humidity–usually close to parallel with segment AB; **11.Point F:** Last weight measure just before midnight.

Continuous hive weight data were considered with respect to average daily weight and within-day changes post treatment. Pre-treatment data were analyzed within the Fall 2016 and Fall 2017 experiments to detect pre-existing group differences and, should any be detected, control for those differences by using pre-treatment parameter values as covariates. Weight data were detrended for each day by subtracting the hive weight estimate at midnight (or closest time thereafter) from each subsequent weight value over the next 24 h (see [28]). The resulting within-day weight datasets were modeled using the “segmented” function in R which fits a segmented line derived from a linear or generalized linear model to a dependent variable using a bootstrapping procedure [29]. Bee colonies outside of a nectar flow during winter tend to lose weight and exhibit consistent daily patterns (Fig 1). Piecewise regressions with 4 breakpoints were fit to the data, which yielded estimates for 10 parameters: 4 break point values, 5 slope values and the adjusted r^2^. Because the data were detrended by subtracting the raw data value at midnight, daily datasets were mathematically independent. A repeated measures MANOVA was conducted on these daily parameter values of interest (letters refer to Fig 1):

1. Dawn break point (Point B);
2. Slope of the first segment after dawn, usually the 2nd segment (Segment BC);
3. Dusk break point (usually the 4^th^ break point, Point E);
4. Night slopes: average slopes of the 1st and 5th segments (Segments AB and EF), which represent hive weight changes during the night;

For statistical analysis, if the 1st break point occurred before 4AM, the 2nd break point was used as the dawn forager departure (with no restrictions placed on that second estimate) and the slope of the 3rd, rather than 2nd, segment was used as the rate of weight loss due to forager departure. Likewise, if the 4th break point occurred after 8PM then the 3rd break point was taken as the dusk forager return (with no restrictions placed on that second estimate). Average parameter values per colony generated by the pre-treatment data set were used as covariates to control for pre-existing differences among treatment groups. Pre-treatment slope values were analyzed with pre-treatment adult bee weight as a covariate.

#### 3. Hive temperature

Internal hive temperature data were divided into daily average values and within-day detrended data. Detrended data were calculated as the difference between the 25 hour running average and the raw data [26]. Sine curves were fit to 3-day subsamples of detrended data taken sequentially by day, and curve amplitudes, representing estimates of daily hive temperature variability, were used as response variables. For hives with two boxes, only temperature data from the lower (brood) box were analyzed. Repeated measures MANOVA (Proc Glimmix, SAS Inc. 2002) was used to evaluate the effects of treatment, day, and their interaction, with the pre-treatment total adult bee mass as a covariate on both the average daily hive temperature and the amplitudes of the fit sine curves. Temperature amplitude datasets were reduced to one value per hive point every 3 d for repeated measures analysis to ensure no overlap between subsamples. As with weight data, average temperature and temperature amplitude data were analayzed pre-treatment as well to detect any pre-existing differences among treatment groups.

## Results

### 1. Hive assessment

Methoxyfenozide treatment did not have a measurable impact on either total adult bee mass (P = 0.73) or brood surface area (P = 0.43) in the Fall 2016 or Fall 2017 experiments, nor were the two experiments different from each other with respect to these metrics (P = 0.36 and P = 0.26, respectively) (Table 1). Considered separately, neither adult bee mass nor brood surface area in the Spring 2018 experiment was affected by treatment (P = 0.14 and P = 0.37, respectively). During the Spring 2018 experiment, two colonies in the low treatment group and one colony in the high treatment group died, in all cases around 1 May. Pre-treatment adult bee mass was significantly correlated with adult bee mass at the 2^nd^ post treatment assessment for the Fall 2016, Fall 2017 and Spring 2018 field experiments (adjusted r^2^ = 0.27, 0.44 and 0.31, respectively). Two colonies in the high treatment group and one in the 125 ppb treatment group died in the Fall 2016 experiment, and one colony in the high treatment group and two in the 125 ppb treatment group died in the Spring 2018 experiment.

Mite fall, as measured using sticky boards, was overall moderate. In the Fall 2016 experiment two hives had mite falls in excess of 40 mites per day. However, those hives survived to the end of the experiment, and among the remaining hives average mite fall per day per treatment group was <10. In the Fall 2017 experiment average mite fall per day per treatment group was <9.

### 2. Patty consumption

Bee colonies in all groups consumed all the pollen feed in the Fall 2016 and Fall 2017 experiments. In the Spring 2018 experiment some colonies did not consume all 1100 g patty but average values (wet weight) among treatment groups were not significantly different when average adult bee mass during feeding period was used as a covariate (P = 0.72): the high treatment group consumed 900±97 g, the low treatment group consumed 868±154 g, and the control group consumed 871±94 g. Total consumption was related to colony size: the average adult bee mass of the colonies that consumed all the patty was 1.61 ± 0.28 kg while that for the colonies that did not was 0.74±0.08 kg, and among colonies that did not finish the patty, consumption was directly proportional to adult bee mass (F_1,10_ = 13.21, P = 0.0046, adj. r^2^ = 0.53).

### 3. Newly emerged bee weights and nurse bee head weights

Log-transformed dry weights of NEBs were not significantly different among treatments from the Fall 2017 experiment through the end of the Spring 2018 experiment (P = 0.13). Methoxyfenozide application did not influence head weight of bees collected in the Fall 2017 experiment. Average head weights (±s.e.) for bees in the high (12.51±0.37 mg), low (12.29±0.31 mg) and control (12.32±0.19 mg) treatments did not differ (P = 1.0).

### 4. HPG size of caged bees

Oral exposure to methoxyfenozide during young adult development did not impact the hypopharyngeal gland sizes of nurse-aged workers (P = 0.31). The average (±s.e.) acinus size of bees exposed to 1000 ppb methoxyfenozide in syrup was 0.021±0.007 mm^2^, while those fed the control treatment had glands that were 0.025±0.007 mm^2^.

### 5. Pesticide analyses

Wax samples taken during the Fall 2016 experiment were analyzed for methoxyfenozide residues and none were found (Limit of Detection, LOD = 1 ppb) with the exception of trace amounts detected in the high treatment in early November, just after the end of the treatment period. For the Fall 2017 experiment, bee bread samples were analyzed rather than wax. In the initial sample, analyzed with respect to a full panel of 192 compounds, only trace amounts of diphenylamine (LOD = 2 ppb) and 118 ppb of thymol were detected (during hive installation, drawn comb obtained from other hives was used). No methoxyfenozide was detected in the bee bread except for samples collected from hives in the high treatment in the November, February and April hive assessments. Those samples contained 5, 2 and 2 ppb methoxyfenozide, respectively. The protein patty samples for the high, low and control treatments were found to have 199, 103 and 0 ppb methoxyfenozide, respectively. These were rounded to 200, 100 and 0 ppb.

### 6. Hive weight for Fall 2016 and Fall 2017 experiments

No significant differences were detected among treatment groups pre-treatment for the Fall 2016 experiment. However, significant differences were observed in the Fall 2017 experiment with respect to the night slope (P = 0.0088), dawn break point (P = 0.0341), and dusk break point (P = 0.0079). In an effort to explain these differences, the distribution of the hive entrance directions among treatments was considered. With respect to the Fall 2016 layout, hives were oriented largely on cardinal directions (N, S, E, W) with at least one hive in each treatment group facing each of those directions. With respect to the Fall 2017 layout, hives facing a northerly direction (NE or NW) were roughly evenly distributed among groups: of the six hives per treatment group, 2 faced north in the control group, 3 in the high treatment group and 4 in the low group. Hives facing an easterly direction were more clumped: 5 faced east in the control group, 3 in the high treatment group and only 1 in the low treatment group. North-south and east-west orientation were then used in further analysis (S1 Table). North-south orientation significantly affected the dawn break point and east-west orientation affected the night slope and the dusk break point.

**Fig 2:**
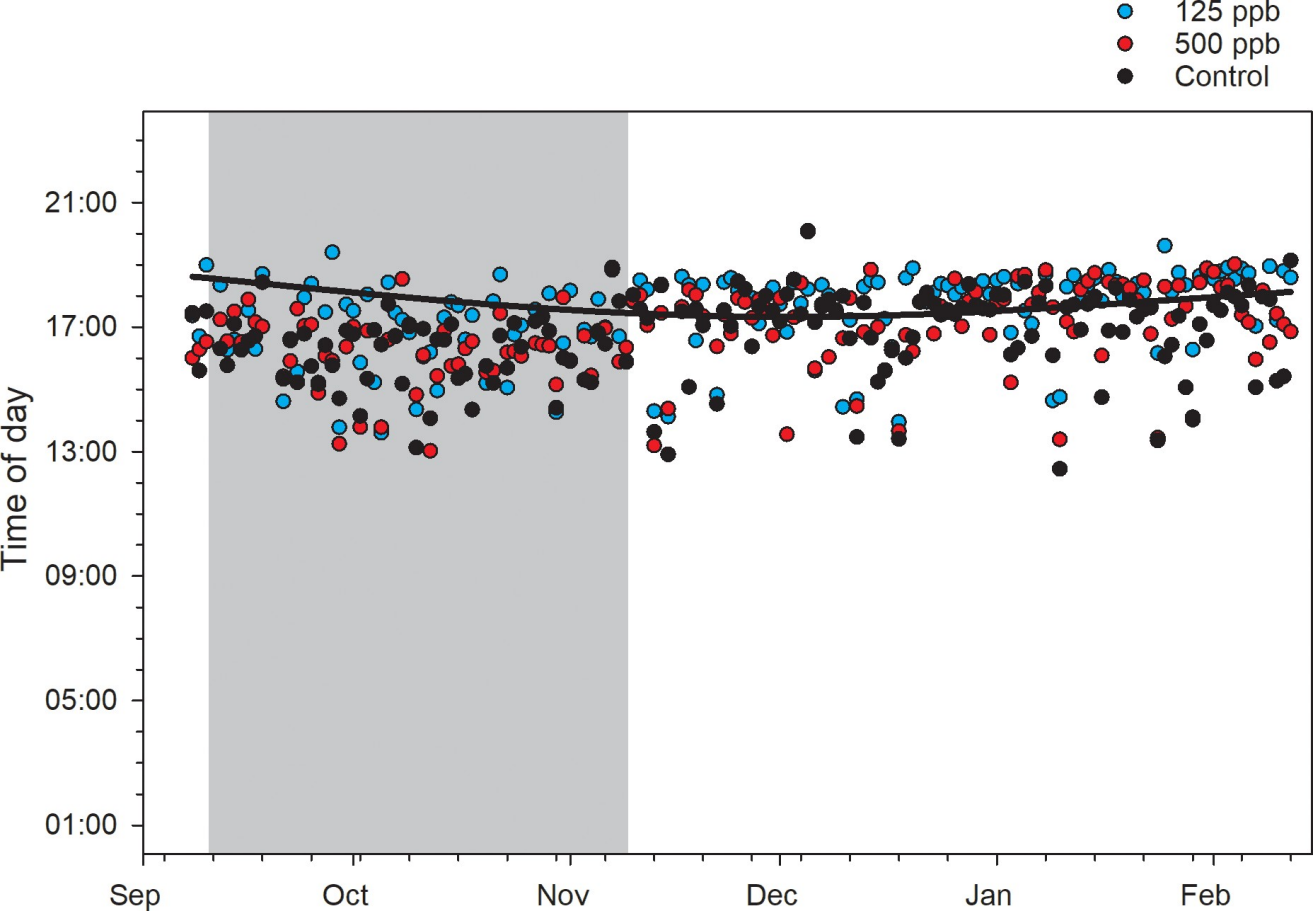
Break points associated with the end of daily activity of bee colonies. The points were obtained using piecewise regression of within-day hive weight changes for the Fall 2017 experiment. Gray area shows the treatment period. Solid black horizontal line shows calculated sunset time.

Piecewise regression curves fit the data well on average. Considering the two Fall experiments separately, average (±s.e.) adj. r^2^ values for the low, high and control groups were 0.94 ±0.03, 0.92±0.05 and 0.91±0.05, respectively, for Fall 2016 and 0.96±0.02, 0.93±0.04 and 0.94±0.03, respectively, for Fall 2017. Dusk break point was significantly affected by treatment even controlling for pre-treatment effects (Fig 2). Post hoc contrasts showed that all treatment groups were significantly different from each other. Lower values in the control group indicate that the dusk break point occurred significantly earlier in the day than for either of the other groups, and earlier for the high treatment group than for the low treatment group. The two fall field experiments were themselves significantly different with respect to dusk break point. Average dusk break point was about 4:39 PM for the control group in Fall 2016, with the high treatment group 20 minutes later and the low treatment group 30 minutes later than control, and dusk was about 4:59 PM for the control group in Fall 2017, with the high treatment group 15 minutes later and the low treatment group 46 minutes later.

**Fig 3:**
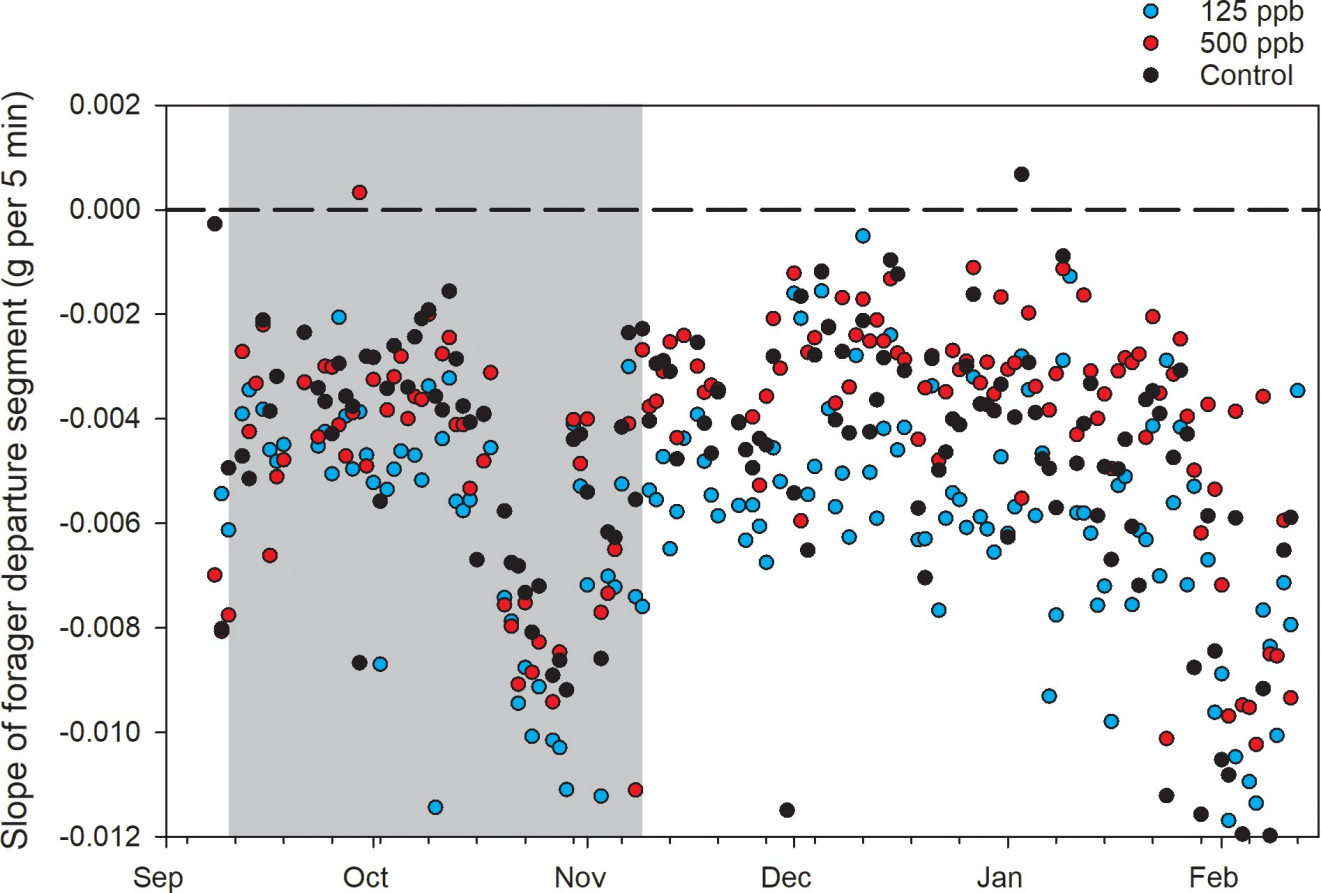
Segment slopes associated with departing foragers for the Fall 2017 experiment. Gray area shows the treatment period. Dashed black horizontal line shows slope = 0.

Slopes of the segments associated with forager departure after the dawn break point were significantly affected by treatment (Fig 3, S2 Table). Post hoc contrasts showed that slopes in the high treatment were significantly shallower, indicating a lower rate of departure, than those in the control group (P = 0.0043). Neither night segment slopes nor dawn break points were significantly affected by treatment (P = 0.51 and 0.34, respectively).

**Fig 4:**
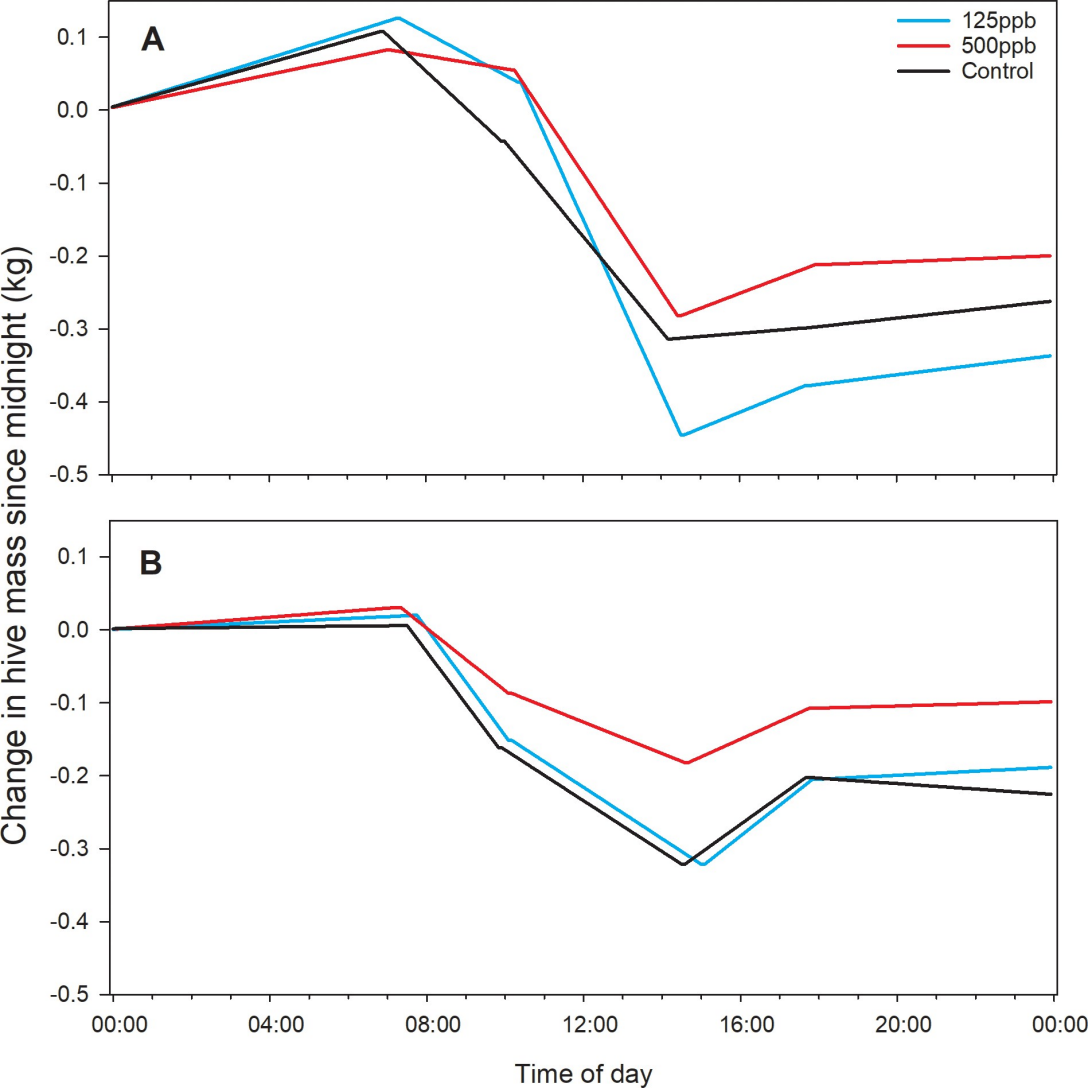
Average within-day weight changes for each treatment group. Curves were estimated using slope and break point values averaged across all colonies within each treatment group and across all days post treatment. (A) Fall 2016 experiment; (B) Fall 2017 experiment. Graphs have the same scales.

Average piecewise regression curves were calculated for each treatment group by averaging slope and break point values across all hives and sample days (Fig 4). Because the post-treatment data were collected in the late fall and winter, with few foraging opportunities, all hives lost weight as the bees consumed food stores. Average slopes at night tended to be positive, probably due to higher ambient relative humidity (the woodenware of the hives can gain weight, as well as any open food cells within the hive). In both years, average curves for the high treatment group were shallower than the control, probably indicating a lower foraging effort.

### 7. Hive weight for Spring 2018 experiment

**Fig 5:**
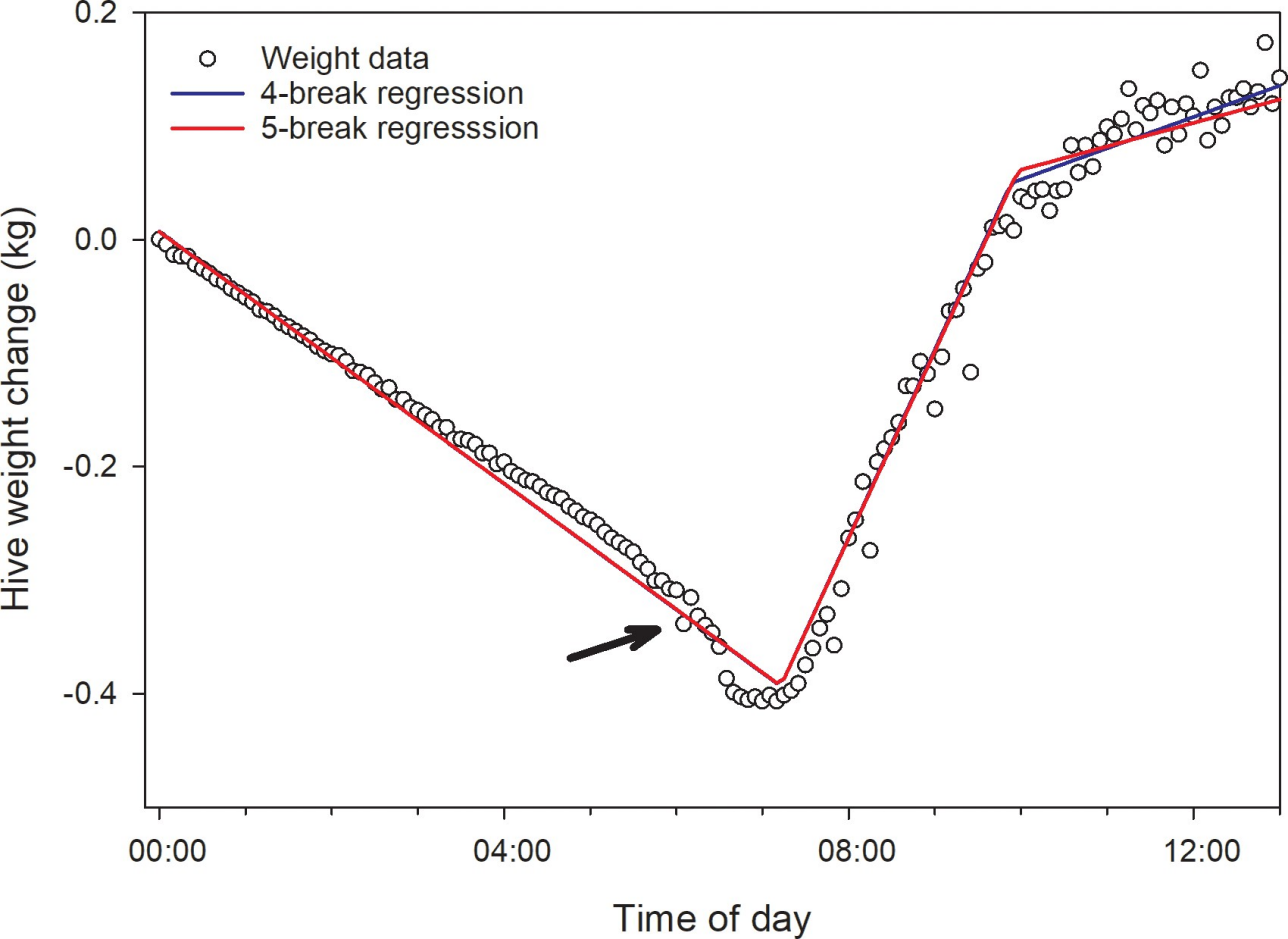
Example of an unsatisfactory piecewise regression curve fit to within-day weight data. Weight data, collected on 29 April 2018 from a hive in the high treatment group, shown with two piecewise regression curve fits: a curve with 4 break points and a curve with 5 break points. Arrow indicates expected “dawn” (1^st^) break point. Note that the 2^nd^ segment, typically associated with forager departure and therefore negative, is in this case positive.

Hive weight consistently decreased during the fall experiments, but average hive weight in all treatment groups increased every day from the end of treatment on 20 April until 6 May. On 7 May average hive in each treatment group started to decrease, indicating the end of a nectar flow, and most hives lost weight after that day until the end of the experiment on 23 May. Within-day hive weight patterns differ depending on whether there is a nectar flow [28] so those two periods (20 April-6 May and 7–23 May) were considered separately. During the nectar flow, only the departing slopes were significantly different among treatments (S3 Table, Table 2). Average slope in the high treatment group was significantly higher than the slopes for either of the other treatment groups. In this case, while average r^2^ ±s.e. values for model fit were high: 0.96±0.03, 0.97±0.03, and 0.91±0.06 for the low, high, and control treatment groups, respectively, visual inspection of the data showed that breakpoints near dawn were not being detected by the algorithm, causing inaccuracies in other segment parameters such as slope values. Increasing the number of break points from 4 to 5 did not appreciably change the overall goodness of fit (average r^2^ = 0.92, 0.97 and 0.96, respectively) and likewise did not improve detection of the break points (Fig 5). These results, therefore, should be subject to verification, and indicate the necessity both of validating the model fit, visually or otherwise, and of improving the curve fit algorithm. No parameters were significantly affected by treatment during the 16 d after the end of the nectar flow.

**Table 2:** Results and treatment means of post hoc comparisons for three response variables with significant treatment effects for 2 analyses conducted on three field experiments on the effects of sublethal methoxyfenozide exposure to honey bee colonies. Times shown are on a 24 h clock; calc. conc. = calculated concentration, temp = temperature; avg = average; ampl. = amplitude. Temperature data in Analysis 1 were restricted to after 30 November in both years.

Analysis	Calc. conc.	Response variables				
		Dawn break point	Dusk break point	Departing slope (g/h)	Temp. avg (°C)	Temp. ampl. (°C)
1. Nov. 2016- Jan. 2017 &	high	7:43±3 a	17:07±4 a	-30.1±12.0 a	20.3±0.2 a	3.0±0.1 a
Nov. 2017- Feb. 2018	low	7:47±3 a	17:28±4 b	-54.0±5.0 ab	21.4±0.2 a	2.5±0.1 ab
	0 ppb	7:41±3 a	16:49±5 c	-73.1±20.1 b	23.1±0.2 a	2.0±0.1 b
2. 20 Apr.-6 May 2018	high	7:00± a	18:28± a	52.6± a	34.4± a	1.0± a
	low	6:44± a	18:45± a	13.2± ab	32.7± a	2.2± a
	0 ppb	6:41± a	18:55± a	-18.0± b	35.8± a	0.5± a

Treatment groups within the same analysis with no letters in common are significantly different at α = 0.05 with a Bonferroni comparison for multiple groups.

### 8. Hive temperature

**Fig 6:**
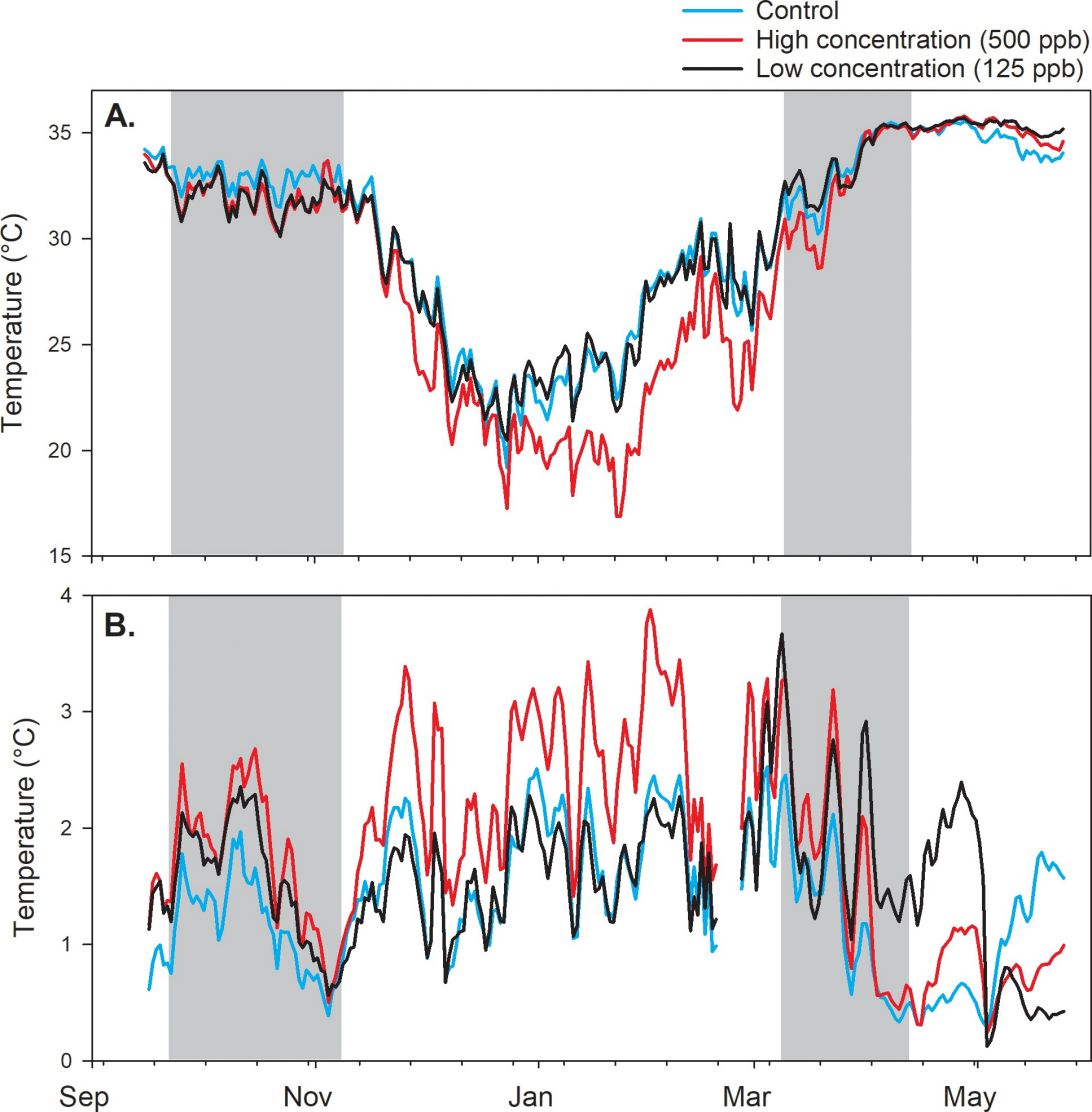
Internal hive temperatures. A) Average temperature for the Fall 2017 experiment through the Spring 2018 experiment; B) Average amplitudes of sine curves fit to internal hive temperature data (temperature variability). Gray zones in A) show the periods of treatment application.

Internal hive temperature was considered with respect to two response variables: 1) average daily temperature; and 2) average temperature variability measured as amplitudes of curves fit to 3-day datasets. Hive temperature has been positively correlated with total adult bee mass, so the pre-treatment value was included as a covariate in all analyses. Regarding pre-treatment temperature average or amplitude, no significant treatment effects were observed (P = 0.78 and 0.82, respectively). Post-treatment, no significant effects were observed when all post treatment data (from end of treatment until final hive assessment), but the low P values (0.06 and 0.07, respectively) suggested there may be trends to explore by sharpening the focus of the analysis. Given that hive temperature is a function of both the bee colony and external ambient conditions, treatment effects may be more likely to be observed when a colony is challenged to manage its temperature. At the beginning of November in both 2016 and 2017 average ambient daily temperatures at the study sites were about 21.7 to 22.8°C. Thirty days later, however, average ambient daily temperatures had dropped to 9.4–12.8°C. Thus, ambient temperatures 30 d after the end of treatment were considered more challenging to the bee colonies and thus more likely to show an effect. Considering only the data from 30 d after the end of treatment until the final hive assessment, treatment effects on average temperature remained not significant (P = 0.06) but significant treatment effects were observed with respect to temperature amplitudes (variability) (S4 Table, Table 2, Fig 6). Temperature amplitudes were lower in the control treatment group (about 1.72°C lower in the Fall 2016 experiment and 2.40°C in the Fall 2017 experiment) than the high treatment group (about 2.55°C in the Fall 2016 experiment and 3.64°C in the Fall 2017 experiment). Neither the control group nor the high treatment group was different from the low treatment group in either experiment.

Neither average internal hive temperatures nor their daily variability were different among treatments either during the nectar flow (P = 0.24 and 0.36, respectively) or after the nectar flow (P = 0.41 and 0.77, respectively) when the February adult bee mass was used as a covariate.

## Discussion

Methoxyfenozide is a preferred insecticide for many crops pollinated by honey bees. One important question regarding sublethal exposure of methoxyfenozide is whether and how such exposure affects bee colonies, such as by reducing brood production or adult bee survivorship, changing colony behavior, or affecting the growth or physiology of individual bees. Methoxyfenozide, with a reported acute oral toxicity of more than 5.0 g per kg for humans, is considered mildly toxic for many vertebrates and crustaceans; with respect to honey bees, it has been reported as “moderately toxic” [10] and “relatively nontoxic” [34]. Most studies on sublethal pesticide exposure rely on invasive hive assessments to estimate adult bee and brood populations. To avoid antagonizing the bees or losing the queen, these assessments are typically carried out about every 4–6 weeks, as was done here, and no effects of methoxyfenozide were observed with respect to hive assessment data (total adult bee mass or brood surface area) in this study. These data are important, but they provide little information on daily or hourly changes in colony-level behavior such as foraging and thermoregulation. In this study we monitored colony behavior using hive scales, to observe hourly weight changes associated with, for example, foraging activity [28], and using temperature sensors to measure themoregulation. Both types of sensors have been successfully used to detect the effects of sublethal exposure of honey bees to a neonicotinoid, imidacloprid [27].

Observed concentrations in the treatment patties were 17.6% lower than the calculated concentration of 125 ppb and 60.2% lower than the calculated concentration of high treatment. Disparities between calculated and observed concentrations of pesticides mixed in pollen patties have been reported at about that magnitude elsewhere [35]. Such disparities may be due to several factors, including insufficient mixing in a heterogeneous material (pollen patty), or a breakdown of the compound due to chemical reactions or biological activity in the patty environment. Using the observed concentration values, the bee bread results suggest that 2–5% of the bee bread sampled was treatment patty. Methoxyfenozide concentration was measured in wax samples during the Fall 2016 experiment, and none was detected in any sample. Wax was sampled to determine if the lipophilic nature of the compound facilitated its spread throughout the hive and that none was detected suggests that any contamination of wax is more likely due to contaminated pollen or nectar. As a dry fine particulate, pollen would easily spread through the hive via air currents and would, being lipophilic, become trapped in the wax matrix. Thymol was the only compound detected in significant concentrations and this was attributed to the use of drawn comb, at the beginning of the experiments, from hives subjected to thymol-based Varroa treatments. In an effort to identify other possible pools of methoxyfenozide in the hive, bee bread was sampled For the Fall 2017 and Spring 2018 experiments, Low concentrations of methoxyfenozide were detected in bee bread from the high treatment group and none in the other groups. The concentrations were apparently stable over time, from the end of the fall treatment in November until the end of the spring treatment the following April.

Two kinds of continuous data were collected in these experiments: internal hive temperature and hive weight. Honey bee colony thermoregulation is crucial for brood rearing [36] and some degree of temperature control is evident among groups of bees even in the absence of brood [31]. Internal hive temperature over time has been linked to changes in colony size and phenology [26, 31]. Sublethal doses of pesticides have been associated with temperature regulation by bees in both field [28] and cage studies [37]. In this study, internal hive temperature was significantly more variable in the 200 ppb treatment group during December and January (months with the lowest ambient temperatures). The difference in variability between the 200 ppb treatment group and the control group was significant, about 1°C on average, while the value for the 100 ppb group fell between the two groups.

Hive weight data, after being detrended by removing the value at midnight from subsequent values for the next 24 h, have common patterns [28]. For example, from midnight until the start of the active period, usually just after dawn, bee flight activity is minimal and hive weight changes largely involve water gain or loss depending on the amount of open nectar and ambient relative humidity. After dawn, weight changes usually become sharply negative, as the daily active period for the colony begins and foragers and other bees leave the hive. Whether hives gain or lose weight during the day depends largely on factors such as the success of the foraging bees. In the late afternoon, hives tend to gain weight as flying bees return to the colony; their return is usually complete about dusk, marking the end of the active period. After that point, hive weight changes are once again largely due to the amounts of hydrophilic materials present and to internal and ambient humidity.

In this study, treatment effects were detected with respect to the slope of the segment associated with forager departure and with the dusk break point. Because these parameters resulted from the fit of single piecewise regression curves, they are not entirely independent from each other but rather reflect fundamental differences in overall daily curve shape. Average curve shapes varied from year to year due to variable ambient conditions, but clear treatment effects were also evident within each year. Colonies fed patty containing 200 ppb methoxyfenozide in the fall had significantly shallower forager departure segments than colonies in the control group, suggesting lower foraging activity and, as with the temperature data, average values for the 100 ppb group fell between the two groups. That both these measurements showed similar relationships among the treatment groups implies a standard dose-response, even at these low concentrations.

The dusk break point is a third response variable that showed significant treatment effects but the relationships among treatment groups were different. Colonies in the 100 ppb group had average delay in the dusk break point of 30 minutes and 47 minutes relative to control in Fall 2016 and Fall 2017, respectively; in the 200 ppb group those delays were 20 and 15 minutes. However, in the Fall 2017 dataset some pre-treatment response variables, i.e. night slope, and dawn and dusk break points, also showed strongly significant pre-existing group differences. Of these only dusk remained significantly different among groups after treatment, even with pre-treatment values as covariates. Different dusk break points among groups may have been due to the direction the hives were facing; an east-west bias was detected between the 100 ppb and control groups after the fact (no north-south bias was detected). Neither pre-treatment differences nor any physical layout bias were detected in the Fall 2016 experiment. Further work is needed to determine the effect of physical layout on continuous weight data.

Analyses of continuous weight break points other than dawn and dusk, or of slopes other than the night slope and initial departure slope, were not included here because of difficulties in interpreting any positive results. Although the curves usually have similar shapes among colonies and over time, not all parts of the curves have a clear interpretation, particularly during forage dearth when forage-related environmental cues are absent.

The Fall 2017 field experiment was continued through the following spring, in order to observe longer-term effects of methoxyfenozide exposure. The spring environment differed from that of the fall in two crucial respects: 1) rising temperatures, longer days and increasingly available forage promote colony growth rather than stasis or decrease as observed in the fall; and 2) a nectar flow was under way during the post-treatment period in the spring but not in either fall experiment. Colonies grew rapidly in the spring in spite of reduced pollen patty consumption, suggesting that alternative food sources played a large role. The effects of methoxyfenozide may thus have been diluted by pollen and nectar flows (see [25]) or the bees, as individuals or colonies, were more effective at detoxification. Three colonies died after the first hive assessment in the spring, one in the 200 ppb treatment and the other two in the 100 ppb treatment. As in the fall, hive assessment data were not significantly different among groups. Continuous weight data segment slopes associated with departing bees were significantly affected, with the 200 ppb hives showing higher slope values (indicating fewer departing bees) than other treatment groups. However, visual comparison of the raw daily data with the curve fits indicated that the algorithm was not sufficiently sensitive to break ponts, reducing our confidence in that result. Regression models with 5 breaks, rather than 4, were fit to the data in an effort to increase the sensitivity of the algorithm, but the quality of the fit was not improved.

No differences were detected with respect to hive assessment data (adult bee mass and brood surface area) or on the level of the individual bee (NEB dry weight and head mass of nurse bees). A laboratory study using a far higher (calculated) concentration of methoxyfenozide, 1000 ppb, failed to show any treatment effects on HPG size. The kinds of colony-level behaviors that were measured, i.e. foraging activity and thermoregulation, can be considered functions of either colony size and age structure, or individual adult bee behavior, or both. Greater foraging activity by one colony compared to another may result from a larger adult bee population with a similar proportion of foragers or from a similar adult bee population with a higher proportion of foragers. Reduced variability in internal hive temperatures may result from the better insulation properties of a tighter bee cluster, from more heat, on average, per bee, or simply from more bees. Although brood levels were not significantly different, the 200 ppb treatment group ranked last for each post-treatment hive assessment in both fall experiments, suggesting that thermoregulation differences were likely (but not definitively) linked to brood levels. The methoxyfenozide exposure had no detectable effect on hive survivorship: of the 18 colonies in the Fall 2016 experiment three died (two in the 200 ppb group and one in the 100 ppb group) while none died during Fall 2017 experiment. Further work is needed to link observed changes in colony behavior to longer-term effects on colony performance and survivorship.

## Conclusions

- Exposure of honey bee colonies to methoxyfenozide in supplement patty at an observed concentration of 200 ppb in the fall reduced the colony foraging population and was associated with poorer colony thermoregulation and lower forager population compared to control colonies.
- Exposure to the colonies did not have a measurable effect on the total adult bee mass, the amount of brood, average newly emerged bee body mass or head weight, and caged bees fed 1000 ppb methoxyfenozide in sugar syrup showed no differences in hypopharyngeal gland size.
- Colonies treated for a 2^nd^ consecutive time the following spring showed fewer differences than the same colonies did in the fall.

## Supporting information

S1 Table Effects of hive direction (direction which the hive faced) on piecewise regressions fit to pre-treatment continuous weight data: Night slope, dawn break point, and dusk break point for the Fall 2017 field experiment (PDF). (PDF)

S2 Table Effects of treatment on dawn break point for piecewise regressions fit to continuous weight data for the Fall 2016 and Fall 2017 field experiments (PDF). (PDF)

S3 Table Effects of methoxyfenozide exposure on departing slopes for piecewise regressions fit to continuous weight data for the Spring 2018 field experiment (PDF). (PDF)

S4 Table The effects of methoxyfenozide exposure on log amplitudes of sine waves fit to detrended continuous temperature data (daily internal hive temperature variation) for the Fall 2016 and Fall 2017 field experiments 30 d after the end of treatment until the final assessment (PDF). (PDF)

S1 File Experimental data (XLSX). (XLSX)
