# Supplementary material for: Exposure to sublethal concentrations of methoxyfenozide disrupts honey bee colony activity and thermoregulation
Source: PLoS One. 2019 Mar 28;14(3):e0204635. doi: 10.1371/journal.pone.0204635 (PMC6438536; doi:10.1371/journal.pone.0204635)
Supplement: S1 Table — (PDF) [file pone.0204635.s001.pdf]

S1 Table. Effects of hive direction (direction which the hive faced) on piecewise regressions fit to pre-treatment continuous weight data: night slope, dawn break point, and dusk break point for the Fall 2017 field experiment. Analyses done with an AR(1) covariance matrix.

Response variable	Effect	Num DF	Den DF	F Value	Pr > F
Night slope	North or South	1	40.98	0.10	0.7571
	East or West	1	41.07	9.75	0.0033
	Day	8	124.7	29.15	<.0001
	February adult bee mass	1	40.92	7.73	0.0082
Dawn break point	North or South	1	60.83	4.95	0.0298
	East or West	1	61.06	0.16	0.6935
	Day	8	125.3	1.34	0.2291
Dusk break point	North or South	1	55.76	0.02	0.8828
	East or West	1	56.02	7.48	0.0083
	Day	8	120.9	3.56	0.0010
