# Supplementary material for: Exposure to sublethal concentrations of methoxyfenozide disrupts honey bee colony activity and thermoregulation
Source: PLoS One. 2019 Mar 28;14(3):e0204635. doi: 10.1371/journal.pone.0204635 (PMC6438536; doi:10.1371/journal.pone.0204635)
Supplement: S2 Table — (PDF) [file pone.0204635.s002.pdf]

S2 Table. Effects of treatment on dawn break point for piecewise regressions fit to continuous weight data for the Fall 2016 and Fall 2017 field experiments. “Year” refers to the year of the experiment. Analyses done with an AR(1) covariance matrix.

Response variable	Factor	Num DF	Den DF	F Value	Pr > F
Dusk break point	Treatment	2	918.8	21.49	<0.0001
	Day	101	2692	8.42	<0.0001
	Treat*Day	202	2661	1.05	0.3230
	Year	1	899.7	15.35	<0.0001
	Treat* Year	2	901.3	1.57	0.2092
	Day* Year	78	2690	6.59	<0.0001
	Pre-treat dusk	1	877.7	5.88	0.0155
Departing slopes	Treatment	2	1006	5.11	0.0062
	Day	101	2695	5.50	<0.0001
	Treat*Day	202	2663	1.76	<0.0001
	Year	1	986.9	2.57	0.1092
	Treat* Year	2	986.6	1.25	0.2857
	Day* Year	78	2693	3.31	<0.0001
	Pre-treat slope	1	959.9	0.04	0.8516
