# Supplementary material for: Exposure to sublethal concentrations of methoxyfenozide disrupts honey bee colony activity and thermoregulation
Source: PLoS One. 2019 Mar 28;14(3):e0204635. doi: 10.1371/journal.pone.0204635 (PMC6438536; doi:10.1371/journal.pone.0204635)
Supplement: S3 Table — (PDF) [file pone.0204635.s003.pdf]

S3 Table. Effects of methoxyfenozide exposure on departing slopes for piecewise regressions fit to continuous weight data for the Spring 2018 field experiment

Effect	Num DF	Den DF	F Value	Pr > F
Treatment	2	27.26	4.13	0.0270
Day	13	168.7	3.60	<.0001
Treat*Day	26	168	1.41	0.1009
February adult bee mass	1	29.63	17.65	0.0002
