# Supplementary material for: Exposure to sublethal concentrations of methoxyfenozide disrupts honey bee colony activity and thermoregulation
Source: PLoS One. 2019 Mar 28;14(3):e0204635. doi: 10.1371/journal.pone.0204635 (PMC6438536; doi:10.1371/journal.pone.0204635)
Supplement: S4 Table — (PDF) [file pone.0204635.s004.pdf]

S4 Table. The effects of methoxyfenozide exposure on log amplitudes of sine waves fit to detrended continuous temperature data (daily internal hive temperature variation) for the Fall 2016 and Fall 2017 field experiments 30 d after the end of treatment until the final assessment. Analysis conducted with AR(1) covariance matrix.

Effect	Num DF	Den DF	F Value	Pr > F
Treatment	2	45.79	5.19	0.0093
Day	26	599.8	24.36	<0.0001
Treat*Day	52	591.8	1.11	0.2771
Experiment	1	47.02	14.69	0.0004
Treat* Experiment	2	48.91	1.63	0.2069
Day* Experiment	14	588	25.47	<0.0001
Pre-treat adult bee mass	1	43.42	1.90	0.1755
